# Pawsitive impact: exploring associations between pet keeping and connection to nature

**DOI:** 10.1038/s41598-026-47211-4

**Published:** 2026-04-03

**Authors:** Matthias Winfried Kleespies, Sebastian Schneider, Julia Delic, Lena Fauerbach, Vanessa Hama-Ali, Fiona Jennifer Herrmann, Isabel Kaiser, Melina Kurzawe, Elena Maria Mathes, Hannah Sandra Neuschwander, Paul Wilhelm Dierkes

**Affiliations:** Department of Bioscience Education and Zoo Biology, Goethe University, Max-von-Laue-Straße 13, 60438 Frankfurt am Main, Germany

**Keywords:** Biophilia, Companion animal, Nature connectedness, Nature relatedness, Urban, Rural, Ecology, Ecology, Zoology

## Abstract

People spend less time in nature, contributing to a decline in connection with nature. Pets or companion animals play a prominent role in many people’s lives, yet quantitative empirical evidence on the relationship between pet keeping and connection to nature remains limited. This study therefore surveyed 2548 participants in Germany to examine associations between keeping a dog, cat, horse, other mammal, bird, fish, or terrarium animal and respondents’ connection to nature. A multiple linear regression model including seven pet types and the covariates age, gender, and place of residence was estimated. In this model, keeping a dog, cat, or horse as well as age, gender, and place of residence were associated with connection to nature. To further examine the role of the three covariates, three separate follow-up models testing moderation by age, gender, and place of residence were conducted. While cat and horse keeping were positive in the initial model, these associations may be attributable to age (cats) and gender (horses), whereas dog keeping showed the most consistent positive association across models. These results provide valuable insights into the associations between pet keeping and connection to nature, particularly highlighting that dog keeping is positively associated with higher levels of connection to nature. Given the observational nature of the data, no conclusions about causality or directionality can be drawn. Further research, ideally using longitudinal or experimental designs, is needed to clarify these associations and explore implications for environmental education and conservation strategies.

**Supplementary Information:**

The online version contains supplementary material available at 10.1038/s41598-026-47211-4.

## Introduction

People who keep pets often develop a strong personal bond with their animals. Pets are seen as friends, companions or family members. Pet keepers in particular believe that the contact and companionship of their pet has a positive effect on them^1^. Numerous studies have now scientifically investigated this pet effect on their keepers^2^ and many studies show a positive effect, even if there is great variability^3^. The positive connection between living with animals and health and well-being is emphasized again and again, particularly in the media^1^.

It has been shown that pets can reduce stress, improve vitality^4^ and increase life satisfaction^5^. Pets can also reduce depression and improve emotional well-being^6,7^. Various review studies have come to the conclusion that both mental and physical health can be improved by pets^8,9^. Animals can also be a way to interact with other people, which has a positive effect on socialization^10,11^.

Besides these positive findings, however, there are also critical studies. For example, there are also studies that have found no influence of pets on mental health^12,13^. The differences between people living with and people without pets could also be attributed to other interaction variables such as age and socioeconomic status^14^. In addition, pets can also pose risks such as infections, injuries or damage to property^15^.

The most popular pets are dogs and cats: in the USA, around 62 million households own one or more dogs and 37 million households own one or more cats^16^. In Europe, more than 350 million pets are kept, including 129 million cats and 106 million dogs^17^. However, despite the popularity of pets and the extensive research in this area, there have so far been few studies investigating associations between pet keeping and personal relationships with nature.

### Connection to nature

The personal relationship that a person has with nature is investigated in a wide variety of research fields and, due to its great importance, the number of publications has increased significantly in recent years^18^. Due to the large number of studies in different fields, there is a whole range of different conceptualizations and no generally accepted definition^19^. Depending on the author, the focus is on the emotional connection to nature^20^, the affective, cognitive, and experiential relationship^21^ or the role of nature in a person’s identity^22^. A particularly popular concept is the Inclusion of Nature in Self by Schultz (2002). In this concept, connection to nature is understood as the personal inclusion of nature in one’s own cognitive representation of the self^23^. Although the concepts of connection to nature differ, various studies have shown that there is a close relationship between them and that they probably measure the same underlying construct^24,25^.

The importance of a connection to nature has been shown in numerous studies. For example, people with an increased connection to nature are more likely to show positive environmental behavior^21,26,27^ and are more willing to protect nature^28,29^. In addition to a large number of individual studies, meta-studies have now also been able to prove the positive effect of connection to nature on the conservation behaviors^30,31^. However, a personal relationship with nature does not only have a positive effect on the relationship with the environment. It has now been shown that an increased connection to nature can have a whole range of positive health effects^32^, such as reducing stress, strengthening the immune system and increasing well-being^33–35^. These effects have been confirmed by different meta-studies^36,37^.

### Connection to nature and animals

People generally perceive animals as an important part of nature^38,39^ and many important concepts of connection to nature include animals^20,21^. It is therefore not surprising that there is also some evidence in the literature that animals can have an influence on the connection to nature. For example, there are studies that have provided evidence that a visit to the zoo can have a positive effect on the connection to nature^40–42^, while other studies have found no effect of a visit to the zoo on the connection to nature^43,44^. The presence of birds, contact with them or a higher diversity of birds may have also a positive effect on the connection to nature^45^. Interaction or contact with animals in their natural habitat can also have a positive effect on connection to nature^46^. On the other hand, there are also studies that have found no effect of dog or cat keeping on connection to nature^47^ or that fear and disgust of animals can even have a negative effect^48^. Pet keeping in childhood also appears to have no effect on later connection to nature^49^, although childhood is considered a particularly sensitive phase for the connection to nature^50^.

### Conceptual considerations: why pet type may matter

The relationship between pet keeping and connection to nature can be discussed in light of the biophilia hypothesis, which assumes that humans have a basic tendency to attend to and affiliate with living beings and life-like processes^51,52^. From this perspective, pets may represent an everyday, emotionally meaningful form of human-animal contact that can express or activate such biophilic tendencies and broaden concern beyond the pet to other animals and nature more broadly^47^. At the same time, connection to nature is not only about liking animals, but also about how nature is integrated into one’s self-concept^23^.

Importantly, pet keeping is highly heterogeneous. Different pet types differ systematically in the typical contexts in which people interact with them and in the extent to which pet keeping is linked to repeated, direct experiences in natural environments. This distinction is relevant because time spent in nature and active engagement with natural environments are among the most robust predictors of connection to nature^53,54^. Some forms of pet keeping may structurally increase the frequency and intensity of nature contact in daily life (e.g., through regular outdoor routines), whereas other forms of keeping pets can take place largely indoors (e.g., aquaria or terraria) and therefore do not necessarily imply direct nature experience.

Based on these considerations, the present study does not treat pet keeping as a single, uniform category, but distinguishes between seven common pet types (dog, cat, horse, other mammal, bird, fish, terrarium animal). These categories represent different forms of everyday human-animal relationships and typical interaction contexts, ranging from pets that often require outdoor activity and repeated nature exposure (especially dogs, and potentially horses) to pets that are more commonly kept indoors and do not require contact with natural environments (e.g., fish and terrarium animals). We expected that pets linked to regular outdoor routines and repeated exposure to natural environments would show stronger positive associations with connection to nature than pets whose keeping is largely independent of nature contact. At the same time, the comparison across multiple pet types allows us to test whether associations reflect a general effect of pets or are specific to certain species-related contexts.

Despite the importance of connection to nature and the prevalence of pets, quantitative evidence on differential associations between specific pet types and connection to nature remains limited. This study therefore aims to investigate the link between connection to nature and pet keeping, and to examine how this relationship varies among different types of pets.

## Results

The descriptive results show that more than a third of respondents kept a pet. Most pet keepers had a cat or a dog. The gender, place of residence and age distribution were similar in the groups (Table 1).

**Table 1:** Descriptive characterization of the sample. When specifying the gender distribution and place of residence, the upper value describes the absolute share, the lower value the percentage share within the group. The number of individual pet groups does not add up to the pet keeper group, as people can keep multiple pets.

	*n*	Gender				Age	Place of residence	
		male	female	diverse	No answer	M ± SD	Big city	Small city/village
Pet keeper	1035	265 25.6%	753 72.8%	8 0.8%	9 0.9%	37.59 ± 16.69	609 58.8%	426 41.2%
Dog	567	153 27.0%	403 71.1%	7 1.2%	3 0.5%	37.98 ± 16.46	325 57.3%	242 42.7%
Cat	441	102 23.1%	335 76.0%	1 0.2%	3 0.7%	38.54 ± 17.16	237 53.7%	204 46.3%
Horse	24	2 8.3%	21 87.5%	1 4.2%	0 0.0%	35.71 ± 16.59	13 54.2%	11 45.8%
Other mammal	90	19 21.1%	70 77.8%	0 0.0%	1 1.1%	33.79 ± 14.88	56 62.2%	34 37.8%
Bird	50	10 20.0%	40 80.0%	0 0.0%	0 0.0%	38.36 ± 17.90	19 38.0%	31 62.0%
Fish	36	12 33.3%	21 58.3%	0 0.0%	3 8.4%	40.06 ± 14.38	15 41.7%	21 58.3%
Terrarium animal	44	13 29.5%	30 68.2%	1 2.3%	0 0.0%	37.57 ± 15.51	23 52.3%	21 47.7%
No pet	1513	549 36.3%	937 61.9%	11 0.7%	16 1.0%	37.83 ± 16.74	1097 72.5%	416 27.5%
Total	2548	814 31.9%	1690 66.3%	19 0.7%	24 1.0%	37.74 ± 16.72	1706 67.0%	842 33.0%

**Fig. 1:**
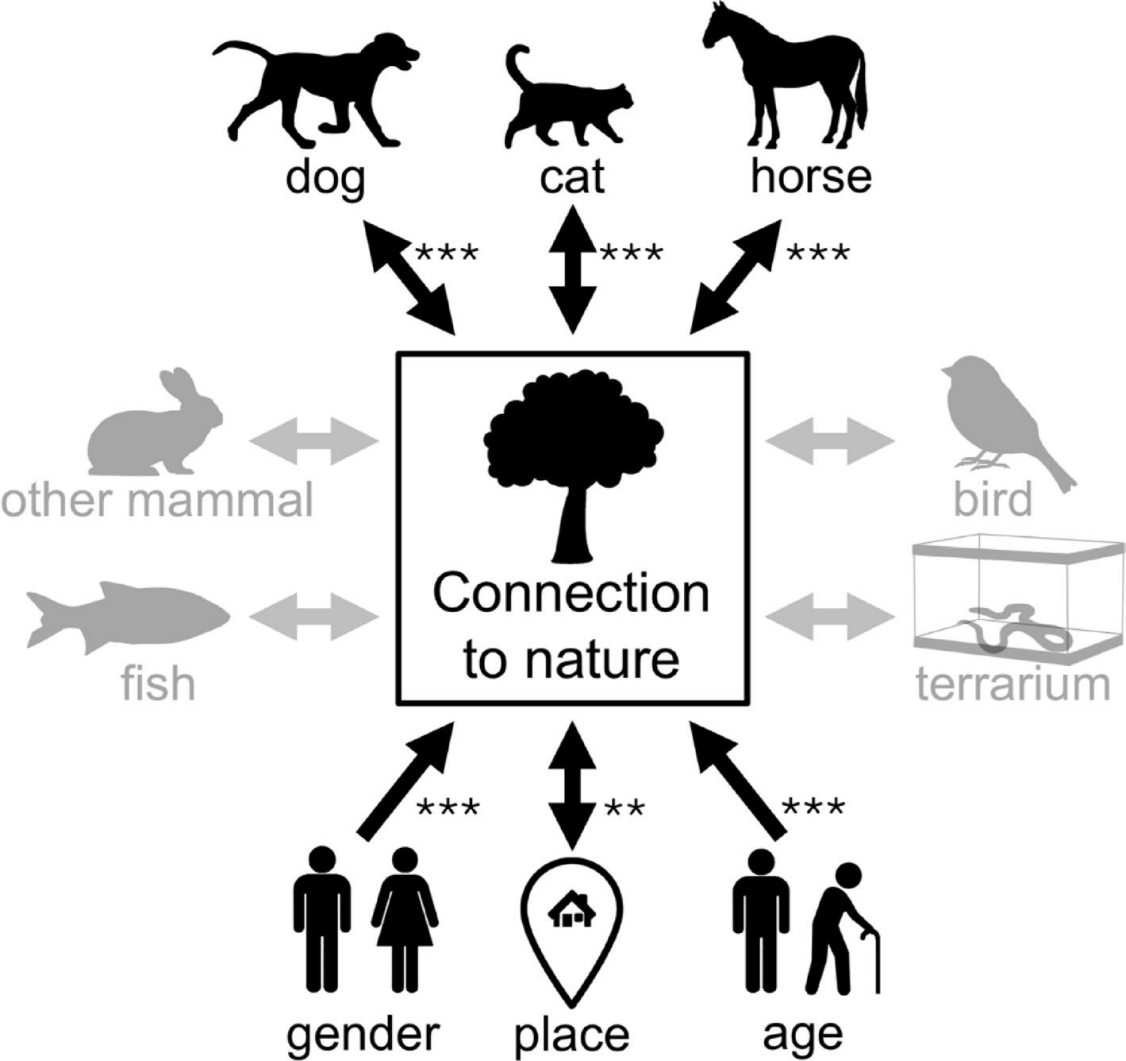
Results of the linear regressions model 1 in which connection to nature was defined as the dependent variable and all other variables as independent variables. Non-significant factors were grayed out. The significance levels are: *p* < 0.05*, *p* < 0.01**, *p* < 0.001***.

The results of the linear regression (Model 1) show that dog keepers, cat keepers and horse keepers have a significantly higher connection to nature than people who do not have pets. The effect for horse keepers is particularly high. In contrast, the effects of other mammals, birds, fish and terrarium animals are not significant. However, the covariates were found to have a significant relationship with the results (Table 2; Fig. 1A).

**Table 2:** Results of the linear regression with all variables (Model 1). Significant changes are highlighted in gray. The intercept refers to a male person who lives in a city and has no pets. The values were rounded to 3 decimal points.

Intercept	Estimate	Std. Error	t-value	*p*-value
	3.155	0.072	43.579	*p* < 0.001
Dog	0.374	0.060	6.278	*p* < 0.001
Cat	0.322	0.066	4.905	*p* < 0.001
Horse	0.892	0.255	3.491	*p* < 0.001
Other mammal	0.195	0.133	1.465	*p* = 0.143
Bird	0.169	0.180	0.942	*p* = 0.346
Fish	0.167	0.218	0.767	*p* = 0.443
Terrarium animal	0.032	0.190	0.171	*p* = 0.864
Place of residence (small city/village)	0.142	0.053	2.654	*p* = 0.008
age	0.021	0.001	14.192	*p* < 0.001
Gender (female)	0.241	0.053	4.586	*p* < 0.001

R² = 0.124, adjusted R² = 0.120, F(10, 2455) = 34.65, *p* < 0.001.

**Fig. 2:**
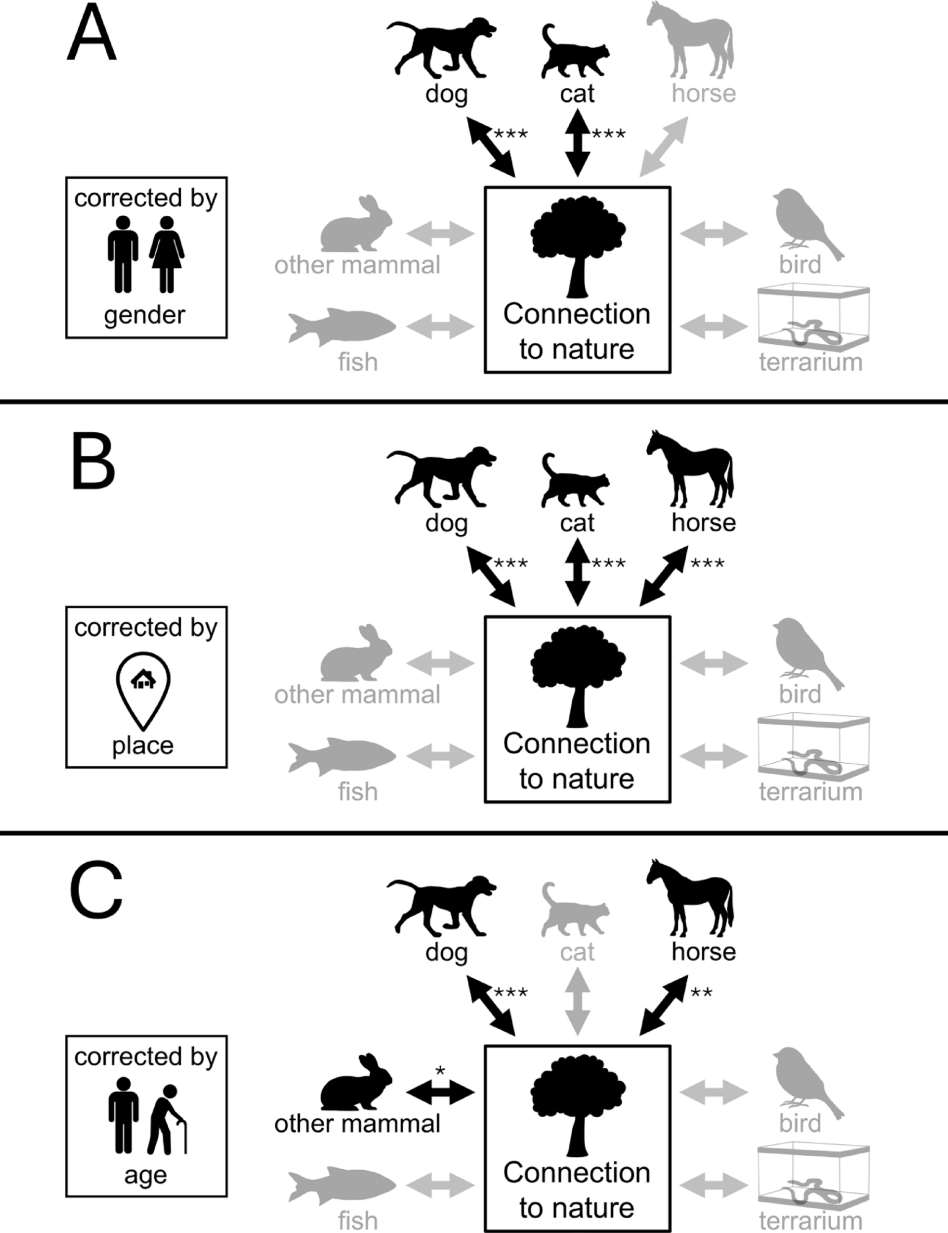
Results of the linear regression Models 2, 3 and 4. (**A**) Model 2 tests whether the association differs by gender. (**B**) Model 3 tests whether the association differs by place of residence. (**C**) Model 4 tests whether the association differs by age. Non-significant factors were grayed out. The significance levels are: *p* < 0.05*, *p* < 0.01**, *p* < 0.001***.

To further examine the role of age, gender, and place of residence, we fitted three separate follow-up moderation models testing interactions with gender (Model 2), place of residence (Model 3), and age (Model 4). Adj. VIF values for all reported models were below 5 (Supplementary Information), indicating that multicollinearity was not a concern in the retained models. Across these models, dog keeping remained positively associated with connection to nature. The positive association of cat keeping observed in Model 1 was not robust when accounting for age (Model 4), while the positive association of horse keeping was not robust when accounting for gender (Model 2). This pattern suggests that the positive associations observed for cats and horses in Model 1 may largely reflect age-related differences between cat keepers and non-keepers and gender-related differences between horse keepers and non-keepers, respectively. In addition, the age interaction model yielded a significant coefficient for other mammals (Model 4, *p* = 0.042). However, this result should be interpreted cautiously given the borderline p-value and the comparatively small size of the other-mammal group (*n* = 90). Place of residence did not materially change the pattern of significance compared to Model 1 (Model 3) (Table 3; Fig. 2).

**Table 3:** Results of three linear models with either gender, place of residence or age as interaction variables. The intercept refers to people without pets. The values were rounded to 3 decimal points.

	Gender (Model 2)		Place of residence (Model 3)		Age (Model 4)	
	Estimate	*p*-value	Estimate	*p*-value	Estimate	*p*-value
Intercept	3.962	*p* < 0.001	4.046	*p* < 0.001	3.363	*p* < 0.001
Dog	0.476	*p* < 0.001	0.410	*p* < 0.001	0.477	*p* = 0.0013
Cat	0.462	*p* < 0.001	0.430	*p* < 0.001	0.015	*p* = 0.923
Horse	0.569	*p* = 0.527	1.469	*p* < 0.001	1.617	*p* = 0.008
Other mammal	0.266	*p* = 0.371	-0.015	*p* = 0.933	0.675	*p* = 0.042
Bird	0.192	*p* = 0.643	0.428	*p* = 0.149	0.247	*p* = 0.563
Fish	0.451	*p* = 0.229	-0.024	*p* = 0.943	-0.186	*p* = 0.767
Terrarium animal	-0.234	*p* = 0.524	-0.067	*p* = 0.804	-0.150	*p* = 0.764
	R² = 0.045, adjusted R² = 0.039, F(15, 2476) = 7.77, *p* < 0.001.		R² = 0.049, adjusted R² = 0.044, F(15, 2520) = 8.73, *p* < 0.001.		R² = 0.118, adjusted R² = 0.113, F(15, 2493) = 22.19, *p* < 0.001.	

## Discussion

The results provide a valuable new perspective on the relationship between pet keeping and connection to nature. The first linear regression model clearly emphasizes the influence of the three covariates age, gender and place of residence. However, the follow-up interaction models suggest that the associations for cats and horses are not robust once age- and gender-related differences are taken into account, whereas dog keeping shows the most consistent positive association across models.

### Dog keeping and connection to nature

In this study, dog keeping showed the most consistent positive link with connection to nature across the regression models. Compared to people without pets, dog keepers reported significantly higher connection to nature.

To understand this association, it is necessary to look at the factors that can influence the connection to nature: A particularly important influencing factor that can have a positive effect on a person’s connection to nature is time spent in nature^53,55^. Even simple walks, for example in the forest or by a body of water, can have a positive effect^21,56^. This can also include artificially created nature in cities, such as urban parks or green spaces^57,58^.

Dogs usually require regular contact with nature, be it through daily walks or activities with the dog, which often take place in areas close to nature, parks or in the forest. It is therefore understandable that the connection to nature of dog keepers is significantly higher compared to people without pets. It is also known that longer, repeated contact with nature or intensive contact with nature can have a positive effect on connection to nature^54,59^. Keeping a dog generally involves this repeated contact with nature, as people are encouraged daily to spend time in nature. This result is consistent with a previous study focusing on dogs and horses, which also found a higher connection to nature for people caring for dogs compared to people without pets^60^.

However, because this study is cross-sectional and based on observational data, no conclusions can be drawn about the direction of the observed association. Selection effects are equally plausible: individuals with a stronger pre-existing connection to nature may be more likely to choose a dog, as dog keeping typically aligns with an outdoor-oriented lifestyle. Future research should therefore test these mechanisms more directly, ideally using longitudinal designs (e.g., before and after dog adoption) and by measuring potential mediators such as frequency and duration of dog walking, use of green spaces, and the quality of engagement with nature. Such approaches would help clarify whether dog keeping contributes to changes in connection to nature or primarily reflects pre-existing differences between groups.

It is also useful to put the estimated effect into context. The estimated difference for dog keeping is around one third of a point on the 7-point Inclusion of Nature in Self Scale (Table 2), which indicates a modest association. Modest effect sizes are common in research on connection to nature. Meta-analyses of interventions and field studies aiming to strengthen connection to nature typically report small-to-medium average changes, even when participants engage in structured nature contact and nature-connection activities^30,61,62^. Therefore, the association observed here should be interpreted as modest, but it may still be meaningful. It should also be noted that the present data were collected exclusively in Germany and primarily in one federal state (Hesse). This geographic scope is important because everyday pet keeping practices and opportunities for nature contact depend on local infrastructure and cultural norms. In Germany, dog keeping is typically linked to outdoor walking and the use of public spaces, which may increase repeated exposure to natural environments. At the same time, access to urban green spaces is comparatively high even in a large city in Hesse such as Frankfurt^63^ and a large part of the population in Germany has green spaces in their direct surroundings^64^. Such conditions may facilitate dog-related outdoor routines even in urban settings and could therefore amplify the association between dog keeping and connection to nature observed here. In contexts where dog keeping is less strongly tied to regular outdoor walking, where access to public green space is more limited, or where cultural meanings and norms around nature and animals differ, the strength and even the direction of associations may vary. Cross-cultural replications are therefore needed before generalizing the present findings beyond the German context.

### Cat keeping and connection to nature

While cat keeping showed a significant association with connection to nature in Model 1, this association was not robust when age was taken into account in the age interaction model (Model 4). This pattern suggests that the positive association observed in Model 1 is largely driven by age differences between cat keepers and non-keepers.

Previous studies have already shown that the connection to nature changes with age: For example, it has been repeatedly found that connection to nature increases especially after puberty^9,65^ and older people engage more with nature^66^. From the results presented here, it can be concluded that it is likely that older people with a higher connection to nature are more likely to be cat keepers than younger people with a lower connection to nature. This means that the link of cat keeping with connection to nature shown in Model 1 is actually due to an age effect.

In contrast to dog keepers, cat keepers are less directly dependent on spending time in nature, as cats are often out independently or kept indoors. It could be argued that animals (like cats) are also part of nature and therefore a higher connection to nature could be expected. For example, people often describe or draw animals when they are asked to draw or describe nature^38,39^. However, this effect does not seem to be sufficient to influence the connection to nature.

Various studies have also found that dog and cat keepers differ in various personality traits. For example, dog keepers are more conscientious, more agreeable and show more extraversion than cat keepers^67,68^. It is therefore possible that dog and cat keepers also differ in other personality traits. People might be more likely to get a dog, for example, if they already have a more positive connection to nature. However, despite the great importance of cats as pets, there is currently a lack of other studies investigating their effects on the connection to nature.

### Horse keeping and connection to nature

Keeping horses was significantly associated with connection to nature in Model 1, but this association was not robust in the gender interaction model (Model 2). Gender effects in the context of connection to nature have been regularly observed in the past: For example, past studies show that women are more likely to care about nature^69^, have a more positive environmental attitude and are more likely to exhibit environmentally friendly behavior^70,71^. An increased connection to nature was also regularly found in favor of women^72,73^, even if not all studies could confirm this trend^74,75^. The fact that the correlation between horse keeping and connection to nature is lost after correcting for gender can therefore be explained by the high number of women who participate in horse riding. For example, almost 80% of equestrians in Germany are female^76^ and female riders also predominate in the sample collected here (> 80%). Therefore, the observed association between horse keeping and connection to nature in this sample might primarily reflect the generally higher connection to nature among women rather than horse keeping itself.

### Other mammals, birds, fish and terrarium animals and connection to nature

Keeping other mammals than cats, dogs or horses was not significantly associated with connection to nature in this study. Studies have shown that contact with large mammals in their natural or semi-natural environment can increase connection to nature^41,77,78^. However, the other mammals examined in this study are mostly small mammals like hamsters, guinea pigs and rabbits. These animals are usually kept in boxes, enclosures or cages in the house or garden. Therefore, activities that could influence connection to nature, such as direct contact with nature or experiencing nature, are not necessary to keep these animals, which may help to contextualize the lack of a significant association with connection to nature.

In addition, the analysis of the data collected here shows that keeping birds also has no significant relationship with connection to nature. This result is surprising at first glance, as studies have repeatedly found the positive effect of bird watching on connection to nature^79–81^. Nevertheless, the observation and keeping of birds differ considerably. Observing birds in the park or in their natural environment is an active process of engaging with nature and spending quality time in nature. In contrast, keeping birds often takes place indoors or in an enclosure, which requires no contact with nature. It is therefore understandable that keeping birds does not automatically influence connection to nature.

A similar result can be observed for the keeping of fish and terrarium animals. Here, too, there is no positive relationship with connection to nature. Past studies have shown that fishing^82^ or diving and looking at animals under water^46^ might increase connection to nature. Direct contact with these animals, for example during gardening or citizen science projects, can also generally improve connection to nature^81,83^. However, similar to birdwatching, it is likely that this is a (social) activity in nature, while keeping these animals alone does not require or imply direct contact with nature.

These findings suggest that apparent discrepancies with parts of the literature may largely reflect differences in (a) the type of human-animal contact studied and (b) methodological approaches. Studies reporting positive associations often focus on nature-based activities and direct encounters in natural environments, whereas keeping some pets can take place largely indoors and therefore does not necessarily increase nature contact. In addition, studies differ in how connection to nature is conceptualized and measured; here, we used the Inclusion of Nature in Self as a brief indicator of perceived self-nature overlap, while other research employs multi-item instruments emphasizing partly different facets of connection to nature^48,49^.

### Place of residence and connection to nature

In Model 1, a significant effect of place of residence on connection to nature was found in the presented data. People in rural areas show a higher level of connection to nature than people in large cities. This result is also consistent with the findings from the literature. For example, the connection to nature decreases in countries with high urbanization rates^84^ and people who tend to live in a rural area have a stronger connection to nature^85^. However, the place of residence interaction model (Model 3) does not lead to any change in significance compared to Model 1, so that it can be assumed in this study that the place of residence plays a minor role.

### Implications for environmental education and conservation psychology

Connection to nature is not only relevant as an outcome in itself, but is also considered an important predictor of pro-environmental behavior and conservation-related engagement^30,31^. Against this background, the present findings matter because they suggest that the association between living with animals and connection to nature is not a general effect, but may depend on keeping contexts that systematically structure everyday opportunities for nature contact. From an environmental education perspective, this suggests that everyday routines such as dog walking may provide frequent, low-threshold opportunities for nature contact, which could help strengthen connection to nature without formal interventions.

#### Limitations

Although this study was conducted with great care, some limitations must be taken into account. The gender distribution of the sample shows that significantly more women than men took part in the survey. This effect is repeatedly observed in social science studies^86^. Although the sample is large enough to make valid statements, this limitation must be kept in mind.

Furthermore, the study is based on a convenience sample, which limits the generalizability of the findings. Participants were recruited through a range of informal channels, including online platforms, university networks, as well as in public spaces such as shopping centers and weekly markets. The survey was distributed as part of a broader research project on human–environment interactions and was not explicitly advertised as focusing on pet keeping or connection to nature. This may have helped reduce topic-specific self-selection bias. Nevertheless, demographic imbalances such as the overrepresentation of younger and female participants could influence both pet keeping patterns and reported levels of connection to nature. These sample characteristics should be considered when interpreting the results.

The distribution between the individual animal groups is also not balanced. While there are large numbers of dog and cat keepers, some groups are comparatively small (e.g., horse keepers *n* = 24, fish keepers *n* = 36, terrarium-animal keepers *n* = 44, bird keepers *n* = 50; Table 1). The potentially limited statistical power resulting from this might reduce the likelihood of detecting small or moderate group differences and results in an uncertainty around the estimates for these categories. In addition, connection to nature was measured using the single-item Inclusion of Nature in Self with a 7-point range, which provides a coarser assessment than multi-item instruments and may further limit sensitivity for subtle differences between smaller groups. In addition, this operationalization is a common environmental-psychological, and thus implicitly anthropocentric, framing: it captures participants’ self-reported perceived relationship to nature rather than relational or multispecies dynamics discussed in parts of the human-animal relations literature. Consequently, non-significant findings for less frequent pet types should be interpreted cautiously. Future studies should therefore employ larger samples for less common pet types and include multi-item and multi-dimensional measures of human-nature connection.

What could also not be taken into account were differences in pet keeping. This is particularly clear in the case of cat keepers: there are pure indoor cats, cats that are occasionally allowed out or pure outdoor cats. There are also differences for other animals: for example, it may makes a difference if somebody keeps a bird in a cage in the living room or has a pigeon loft. It is also possible, for example, that there is a dog in the family, but only certain family members take it for walks, while all family members identify themselves as pet keepers. These individual factors could not be considered in the study.

In addition, this analysis cannot clarify with certainty whether people were already more connected to nature before they bought their pet. It is therefore possible that people with a higher connection to nature tend to get a dog. Another study with a small sample suggests that connection to nature increases after adopting a dog^87^. Nevertheless, a potential influence of previous connection to nature cannot be ruled out.

Despite these limitations, the study offers meaningful insights into the relationship between pet keeping and connection to nature. Even though the sample is not representative, the diversity of respondents and pet species allows for valuable reflections on how everyday human-animal relationships may relate to people’s engagement with nature.

## Conclusion

This study provides new insights into the relationship between pet keeping and connection to nature, an important predictor of pro-environmental behavior and well-being. The results show that dog keeping is the most consistently associated pet type with higher connection to nature. While cat and horse keeping were positive in the initial covariate-adjusted model, follow-up analyses suggest that these associations are largely attributable to age (cats) and gender (horses), whereas dog keeping remains robust across models. These results highlight that dog keeping, in particular, is more consistently associated with higher levels of connection to nature compared to keeping of other pet types. This may point to differences in how individuals engage with their pets and the environments in which these interactions typically occur. However, it is also possible that individuals who already feel more connected to nature are more likely to choose a dog as a companion, highlighting the need for further research to explore the directionality of this relationship.

The observed association between dog keeping and connection to nature points to the importance of everyday relationships in shaping how people engage with the natural world. Whether dog keeping encourages more time in nature or reflects an already existing connection to it, the findings suggest that animals can play a meaningful role in how individuals experience or express their relationship with nature.

At the same time, the absence of similar associations for other animals suggests that the relationship between pet keeping and connection to nature likely varies depending on the species and context of keeping. However, the results should be interpreted with caution, as possible influencing factors such as personality or the social environment were not taken into account in this study. Further research is needed to investigate underlying factors and to better understand the mechanisms behind the relationship between pet keeping and connection to nature.

## Methods

### Participants and procedure

In total, data were collected from 2589 adults in Germany (66.3% female, 31.8% male, 0.8% diverse, 1.0% no answer). We used a convenience sampling approach. Participants were recruited via multiple channels, including university and community mailing lists and personal networks, as well as paper-pencil distribution in public spaces (e.g., weekly markets and shopping streets) in the Frankfurt am Main region (Taunus and Odenwald; Hesse, Germany). This mixed recruitment strategy was chosen to reach a heterogeneous group of respondents beyond a single setting. Respondents were required to be based in Germany at the time of participation. This restriction reflects the study’s regional focus and helped to avoid additional cross-national heterogeneity. Questionnaires for which the place of residence could not be assigned or for which the place of residence was outside Germany were discarded (*n* = 41). The final data set for analysis contained 2548 questionnaires. Some data sets were incomplete, but were retained because not all variables were required for all models. The survey period ran from October 2023 to October 2024. The study was not designed to yield a nationally representative sample of pet keepers in Germany. The results should therefore be interpreted as evidence from a large convenience sample of adults in Germany, with limited generalizability beyond this context.

### Structure of the survey instrument

At the beginning of the questionnaire, there was a short information text informing the participants about the content and objectives of the study and the duration of the survey (approximately 10 min). The voluntary nature of participation was also emphasised. Basic demographic data such as age in years, gender and the zip code of the place of residence were collected. For the following analysis of the place of residence, 2 groups were formed based on the postcodes: People in large cities (> 100,000 inhabitants) and people in cities/villages (< 100,000 inhabitants). In order to determine pet keeping, respondents were asked whether they keep a pet. If this was the case, a text field was used to indicate which pet it was.

### Measuring connection to nature

To measure connection to nature the Inclusion of Nature in Self Scale by Schultz (2002)^23^ was used. This is a single item measurement instrument consisting of seven pairs of circles. One of the circles is labelled ‘nature’ and the other ‘self’. The seven pairs of circles differ in their degree of overlap: from two completely separate circles (1 = very little connection to nature) to two completely overlapping circles (7 = one with nature). The respondent must select the pair of circles that best describe their own relationship with nature. Due to the simplicity of the scale, it is suitable for research in different contexts, such as international studies^84^. The reliability of the scale has now been confirmed despite its one-dimensionality^88^.

The Inclusion of Nature in Self captures the perceived overlap between self and nature as a component of environmental identity^23^. In addition, due to its shorter length, the Inclusion of Nature in Self is particularly suitable for ad hoc surveys, where people often have little time (or motivation) to complete longer questionnaires. Importantly, previous work has shown that the Inclusion of Nature in Self demonstrates acceptable psychometric performance and convergent validity with other established measures of human-nature connection despite its single-item format^24,25^. At the same time, because the present study aimed to detect comparatively modest differences between pet-keeping groups, we acknowledge that a single-item measure with a 7-point response range may provide a less sensitive and less differentiated assessment than multi-item and multi-dimensional instruments (e.g., connectedness to nature, nature relatedness, environmental identity).

### Analysis

All statistical analyses were conducted in R version 4.4.1. In order to process the data, a binary classification (0 = non-keeper, 1 = keeper) was made for each person as to whether they were the keeper of at least.

- A cat (person stated that they keep at least one cat),
- A dog (person stated that they keep at least one dog),
- A horse (person stated that they keep a horse or were part of a riding partnership),
- Another mammal (person stated that they keep at least one mammal that was not a dog, cat or horse, e.g. rabbit or hamster),
- A bird (person stated that they keep at least one bird),
- A fish (person stated that they keep fish) or.
- A terrarium animal (person stated that they keep an animal living in a terrarium such as a reptile, amphibian or invertebrate).

If participants reported more than one pet type, they were assigned to multiple categories accordingly. The mapping was based on explicit animal names (including common spelling variants) and the above-mentioned categories. Because the animal names were clear and coding followed fixed rules, we did not calculate inter-rater reliability.

To analyze the relationship between pet keeping and connection to nature, a series of multiple linear regression models were employed. In the first step, a multiple linear regression model (Model 1) was created with connection to nature as the dependent variable, the seven pet categories as predictors, and age, gender, and place of residence as covariates. To explore whether associations between pet keeping and connection to nature differed by gender, place of residence, or age, we then fitted three separate moderation models including the respective interaction terms: pet keeping and gender (Model 2), pet keeping and place of residence (Model 3), and pet keeping and age (Model 4). Robust standard errors were applied to account for potential deviations from homoscedasticity.

Model diagnostics were performed for residual analysis to assess the assumptions of linear regression. Residuals of the models were plotted and visually inspected to check for normal distribution. As the residuals appeared normally distributed and no obvious violations of homoscedasticity or independence were observed, it was assumed that the assumptions for linear regression were satisfied. Adjusted variance inflation factors (adj. VIF) were calculated to assess multicollinearity. Values below 3 are generally considered unproblematic, while values above 5 may indicate potentially problematic multicollinearity^89^. Adj. VIF values for all retained models are reported in the supplementary information. These values were predominantly below 3 and did not exceed 5, indicating no concerning multicollinearity in the reported models. For each regression model, model fit and overall significance were assessed by reporting the coefficient of determination (R²), the F-statistic, and its corresponding p-value.

Because some pet categories were comparatively small (e.g., horse keepers *n* = 24, fish keepers *n* = 36, terrarium-animal keepers *n* = 44, bird keepers *n* = 50; Table 1), we conducted an a priori sensitivity analysis using GPower^90^ to quantify which group differences could be detected with the available sample sizes. Using conventional assumptions for two-sided group comparisons (Means; α = 0.05; power = 0.80) with respondents without pets as the reference group (*n* = 1513; Table 1), the minimum detectable standardized mean differences were small for dog (d = 0.13) and cat keepers (d = 0.15), but larger for less common pet types (other mammals: d = 0.31; birds: d = 0.41; terrarium animals: d = 0.44; fish: d = 0.48; horses: d = 0.59). This indicates that while the study was well powered to detect small differences for the most common pet types, null findings for rarer pet categories should be interpreted cautiously because only moderate or larger differences would have been detectable in these groups.

## Supplementary Information

Below is the link to the electronic supplementary material.

Supplementary Material 1

Supplementary Material 2

## Data Availability

The data that support the findings of this study were archived on a password-protected server at Goethe University and are available on request from the corresponding author. The data are not publicly available due to local guidelines and data protection regulations of the ethics committee.
