# Supplementary figures and images for: Pawsitive impact: exploring associations between pet keeping and connection to nature
Source: Sci Rep. 2026 Apr 3;16:11381. doi: 10.1038/s41598-026-47211-4 (PMC13049069; doi:10.1038/s41598-026-47211-4)

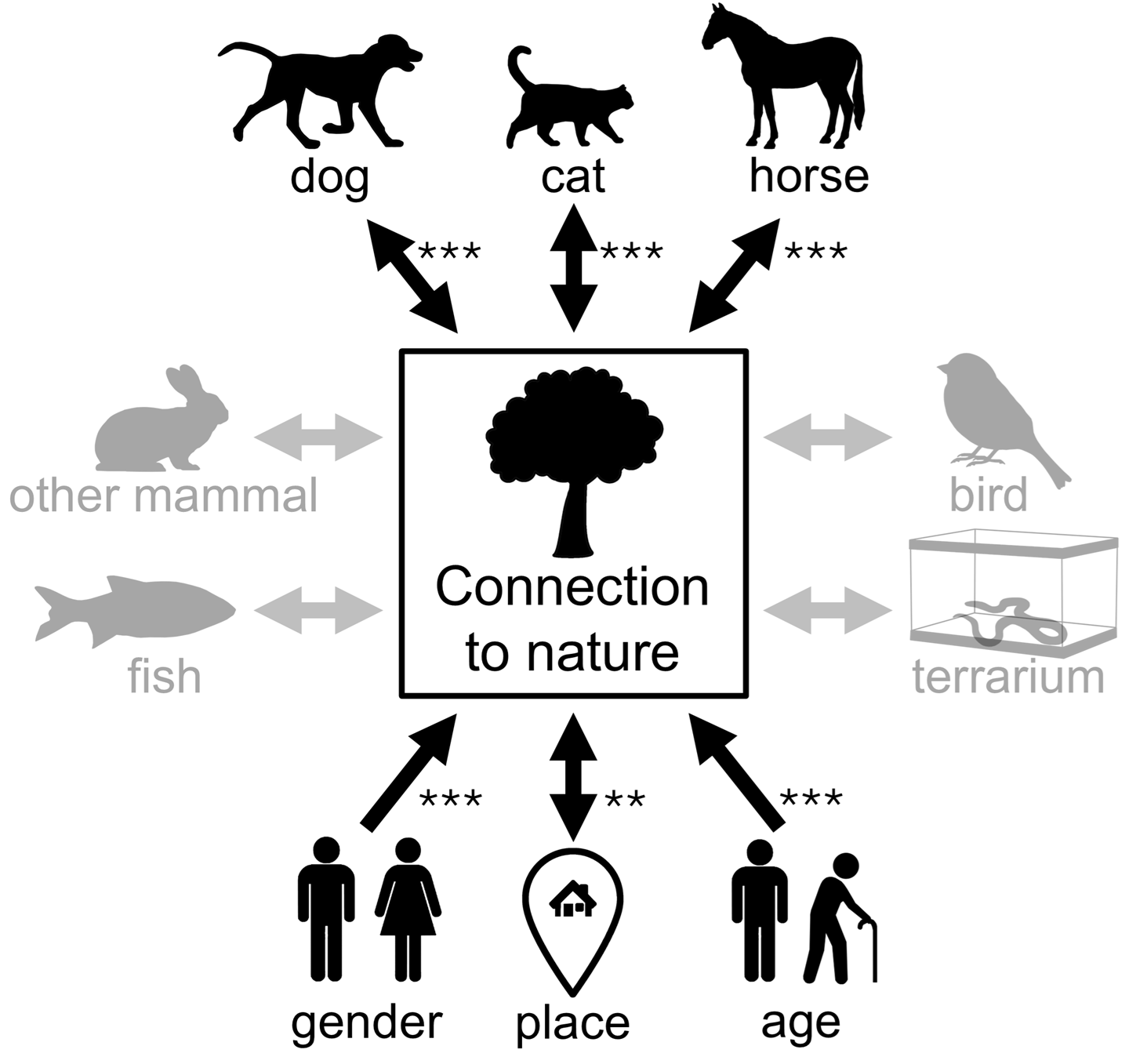

Supplement: Supplementary file 1 — Supplementary Material 1 [file 41598_2026_47211_MOESM1_ESM.png]
