# Supplementary material for: Pawsitive impact: exploring associations between pet keeping and connection to nature
Source: Sci Rep. 2026 Apr 3;16:11381. doi: 10.1038/s41598-026-47211-4 (PMC13049069; doi:10.1038/s41598-026-47211-4)
Supplement: Supplementary file 2 — Supplementary Material 2 [file 41598_2026_47211_MOESM2_ESM.docx]

**Supplementary Material**

Original Article

**Pawsitive impact: Exploring associations between pet keeping and connection to nature**

This supplementary file reports the adjusted variance inflation factors (adj. VIF) for all retained regression models

VIF Model 1

| Term | Adj. VIF |
| --- | --- |
| Dog | 1.01 |
| Cat | 1.02 |
| Horse | 1.00 |
| Other mammals | 1.01 |
| Birds | 1.03 |
| Fish | 1.03 |
| Terraristic | 1.02 |
| Place | 1.03 |
| Age | 1.01 |
| Gender | 1.01 |

VIF Model 2

| Term | Adj. VIF |
| --- | --- |
| Dog | 1.89 |
| Cat | 2.03 |
| Horse | 3.39 |
| Other mammals | 2.16 |
| Birds | 2.26 |
| Fish | 1.69 |
| Terraristic | 1.88 |
| Gender | 1.22 |

VIF Model 3

| Term | Adj. VIF |
| --- | --- |
| Dog | 1.30 |
| Cat | 1.34 |
| Horse | 1.36 |
| Other mammals | 1.27 |
| Birds | 1.62 |
| Fish | 1.56 |
| Terraristic | 1.39 |
| Place | 1.30 |

VIF Model 4

| Term | Adj. VIF |
| --- | --- |
| Dog | 2.51 |
| Cat | 2.48 |
| Horse | 2.42 |
| Other mammals | 2.51 |
| Birds | 2.41 |
| Fish | 3.05 |
| Terraristic | 2.68 |
| Age | 1.25 |
